# Optimistic update bias increases in older age

**DOI:** 10.1017/S0033291713002602

**Published:** 2013-11-04

**Authors:** R. Chowdhury, T. Sharot, T. Wolfe, E. Düzel, R. J. Dolan

**Affiliations:** 1 Institute of Cognitive Neuroscience, University College London, UK; 2 Wellcome Trust Centre for Neuroimaging, Institute of Neurology, University College London, UK; 3 Department of Cognitive Perceptual and Brain Sciences, University College London, UK; 4 Otto-von-Guericke University Magdeburg, Institute of Cognitive Neurology and Dementia Research, Magdeburg, Germany; 5 German Centre for Neurodegenerative Diseases (DZNE), Magdeburg, Germany

**Keywords:** Ageing, anterior cingulate cortex, optimism

## Abstract

**Background:**

Healthy older adults report greater well-being and life satisfaction than their younger counterparts. One potential explanation for this is enhanced optimism. We tested the influence of age on optimistic and pessimistic beliefs about the future and the associated structural neural correlates.

**Method:**

Eighteen young and 18 healthy older adults performed a belief updating paradigm, measuring differences in updating beliefs for desirable and undesirable information about future negative events. These measures were related to regional brain volume, focusing on the anterior cingulate cortex (ACC) because this region is strongly linked to a positivity bias in older age.

**Results:**

We demonstrate an age-related reduction in updating beliefs when older adults are faced with undesirable, but not desirable, information about negative events. This greater ‘update bias’ in older age persisted even after controlling for a variety of variables including subjective rating scales and poorer overall memory. A structural brain correlate of this greater ‘update bias’ was evident in greater grey matter volume in the dorsal ACC in older but not in young adults.

**Conclusions:**

We show a greater update bias in healthy older age. The link between this bias and relative volume of the ACC suggests a shared mechanism with an age-related positivity bias. Older adults frequently have to make important decisions relating to personal, health and financial issues. Our findings have wider behavioural implications in these contexts because an enhanced optimistic update bias may skew such real-world decision making.

## Introduction

Increasing age usually heralds an array of negative life events including bereavement, reduced social networks, a decline in physical health and cognitive function, together with an inevitable time horizon foreshortening (Rowe & Kahn, 1987; Hedden & Gabrieli, 2004). Viewed from the perspective of young adulthood, a reasonable inference might be that this should portend an increasing pessimism. Yet older adults have higher levels of emotional well-being than their younger counterparts, including a decline in their experience of negative emotions (Blanchflower & Oswald, 2008; Carstensen *et al.* 2011; Stone *et al.* 2010).

In general, many studies show an age-related ‘positivity effect’ on cognitive processing (for reviews, see Mather & Carstensen, 2005; Isaacowitz & Blanchard-Fields, 2012). For example, in comparison to their younger counterparts, older adults remember faces displaying positive emotions more than those displaying negative emotions (Charles *et al.* 2003), have less rich autobiographical memory for negative events (Comblain *et al.* 2005) and experience less negative arousal when anticipating monetary loss (Samanez-Larkin *et al.* 2007). Such findings have been interpreted within the framework of socio-emotional selectivity theory, whereby changing time horizons may lead to modification and prioritization of emotionally relevant goals (Carstensen *et al.* 1999; Charles & Carstensen, 2010). An alternative account suggests that positivity may arise serendipitously as a consequence of selective age-related neurodegeneration (Cacioppo *et al.* 2011).

Few studies have addressed the effect of age on optimism and the results are inconsistent. Optimism has been defined as the tendency to overestimate future positive events and underestimate future negative events (Weinstein, 1980). One such study showed that older adults had a more optimistic style when explaining life events (Isaacowitz, 2005) whereas another found that younger, rather than older, adults had a more optimistic outlook about the future (Lachman *et al.* 2008). A series of studies have investigated optimism in young individuals, identifying an asymmetry whereby beliefs about future negative events are updated more in response to better than expected (‘desirable’) information than to worse than expected (‘undesirable’) information (Sharot *et al.* 2011, 2012*a*,*b*).

Optimism in younger adults seems to be related, at least in part, to functional activity of the inferior frontal gyrus (Sharot *et al.* 2011) and the anterior cingulate cortex (ACC) (Sharot *et al.* 2007). Importantly, a large body of literature links age-related functional magnetic resonance imaging (MRI) differences in the ACC to a positivity effect on cognitive processing and greater emotion regulation with age (Kensinger & Schacter, 2008; Leclerc & Kensinger, 2008; Brassen *et al.* 2011, 2012; Samanez-Larkin & Carstensen, 2011). Structural abnormalities of the ACC, in particular the dorsal ACC, have been identified in clinical depression, where pessimism is a core feature (Vasic *et al.* 2008; Pizzagalli, 2011). A greater volume of the dorsal, as opposed to the ventral, subregion has been found in healthy individuals who show greater cognitive reappraisal, a putative mechanism for regulation of emotion (Giuliani *et al.* 2011). Although ageing is associated with volumetric changes in frontal brain structures (Good *et al.* 2001; Hedden & Gabrieli, 2004), the relationship between ACC volume and positivity in older age is unknown.

**Fig. 1.**
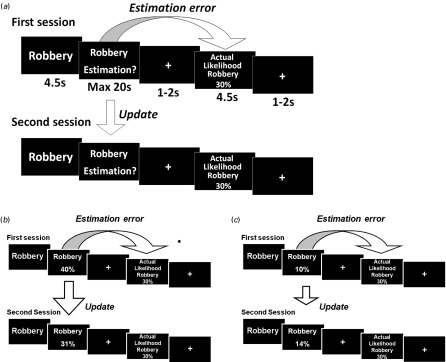
Task design. (*a*) On each trial, participants were presented with one of 45 adverse life events and asked to estimate how likely this event was to occur to them in the next 5 years. They were then presented with the average probability of that event occurring to a person similar to themselves in the same sociocultural environment. For each event an estimation error was calculated as the difference between the participants' estimation and the average probability provided. The second session was the same as the first session. (*b*) For each event, an update was calculated as the difference between the participants' first and second estimations. If the participants' first estimate was higher than the average probability provided, that trial was classified as ‘desirable’ because the information presented was better than expected, calling for an adjustment in an optimistic direction. (*c*) If the participants' first estimate was lower than the average probability provided, that trial was classified as ‘undesirable’ because the information presented was worse than expected, calling for an adjustment in a pessimistic direction.

To determine whether optimistic and pessimistic belief formation about the future is altered in older age, we tested young and older healthy adults using a modified version of a previously described belief updating paradigm (Sharot *et al.* 2011) (Fig. 1). In addition, to determine whether the volume of the ACC was related to age-related differences in updating beliefs, we used structural neuroimaging and a region of interest (ROI) analysis of the dorsal and ventral subregions together with an independent whole-brain analysis to identify the associated structural neural correlates.

## Method

### Participants

Eighteen younger adults (mean age = 22 years, s.d. = 2.29) and 18 older adults (mean age = 66 years, s.d. = 5.62), all of whom were healthy and not depressed, participated in this study (see Online Supplementary Table S1). Participants were recruited through an advertisement placed in a local newspaper and by word of mouth. Written informed consent was obtained from all participants. The study received ethical approval from the University College London (UCL) research ethics committee. Older participants were screened using standardized neuropsychological tests to ensure their global cognitive function was within the normal range (see Supplementary Method).

### Belief updating task

As shown in Fig. 1, we used a modified version of the task by Sharot *et al*. (2011). The task structure remained the same and can be summarized as follows: participants viewed 45 negative events, such as robbery (4.5 s) (see Supplementary Material for a list of all stimuli), and were asked to type on a keyboard their estimate of how likely the event was to occur to them in the next 5 years (20 s). After a brief fixation cross (1–2 s), they were presented with the average probability of this event happening to someone in the general population (4.5 s), followed by a further fixation cross (1–2 s). Immediately after this first session, participants performed the same task again. We adapted the task to make it applicable to both young and older adults as follows. First, all participants were given longer to input their responses than on previously run versions of this task. We note that reaction times did not differ between young and older adults (see Supplementary Results and Table S2). Second, we excluded health-related stimuli as they are known to have very different probabilities across age. Third, participants were asked to rate how likely events were to occur to them in the next 5 years to account for differences between time perspectives in the two groups. At the end of the study we asked participants to estimate their lifespan; all participants thought they would live longer than 5 years, ensuring that they could imagine the events occurring in their lifetime.

### Main behavioural analysis

#### Belief updating task

The key test in this study was to determine differences between young and older adults pertaining to changing their beliefs after being presented with information that was better or worse than expected. For each subject, each trial was classified as ‘desirable’ or ‘undesirable’ depending on whether their initial estimate was higher or lower than the average statistic respectively. Thus, although all trials involved negative events, participants could receive desirable (better than expected) or undesirable (worse than expected) information for each event. We then calculated their change in beliefs (‘update’) as the difference between their first and second estimation (first estimation minus second estimation for desirable trials; second estimation minus first estimation for undesirable trials). We could then examine whether the update differed between desirable and undesirable trials, indicating an ‘update bias’, and whether age affected this bias. For this analysis of update, we used a repeated-measures ANOVA with valence (desirable/undesirable) as the within-subjects measure and age group (young/older) as the between-subjects measure. To account for potential confounding variables, the following were included as covariates in this analysis: differences on all subjective rating scales, memory errors, first estimation error (i.e. a measure of the initial difference between the subject's own estimate and the probability given; see Table S3), the number of desirable and undesirable trials, reaction times, IQ and Life Orientation Test – Revised (LOT-R) scores (a measure of trait optimism).

#### Memory test and subjective rating scales

After the belief updating task, participants completed a self-paced memory test in which they were asked to recall the average probabilities that were presented previously for all events. Memory errors were calculated as the absolute difference between the average probability presented previously and the participants' recollection of that statistical number. Participants also rated all 45 events on the following subjective measures using a Likert scale from 1 (not at all) to 6 (very): vividness, familiarity, personal experience, emotional arousal and negativity (Table S4). Subjective ratings for each measure and memory errors were analysed using a repeated-measures ANOVA with valence (desirable/undesirable) as the within-subjects factor and age group (young/older) as the between-subjects factor. We also calculated a difference measure between subjective rating scores for desirable and undesirable trials to include as covariates in the main behavioural analysis.

#### Trait optimism

Participants completed the LOT-R, which provides a measure of trait optimism (Scheier *et al.* 1994). Scores range from 0 (pessimistic) to 24 (optimistic). Between-age-group differences in trait optimism were compared using an independent *t* test. As LOT-R scores were higher in older adults, we also included LOT-R scores as a covariate in the main analysis of updating of beliefs based on desirable or undesirable information.

### Behavioural statistical analysis

All analyses were conducted using SPSS version 17.0 (SPSS Inc., USA). The significance level for the ANOVAs was set at *p* < 0.05, two-tailed. We did not perform corrections for multiple comparisons when testing for *post-hoc* differences in memory performance and subjective ratings because the aim of these analyses was to identify potential confounding factors that could be added as covariates to our main behavioural analysis. Thus, by not using Bonferroni corrections, our analyses were more stringent.

### Neuroimaging acquisition

A high-resolution structural MRI data set was acquired on a 3.0-T Trio MRI scanner (Siemens, Germany) using a 32-channel head coil (see Supplementary Method for full details of the sequence protocol). T1-weighted (T1w) images were segmented into grey matter, white matter and cerebrospinal fluid (Ashburner & Friston, 2005) using the New Segment toolbox in SPM8 (http://www.fil.ion.ucl.ac.uk/spm/). Using a diffeomorphic registration algorithm (DARTEL), the grey matter maps were warped to a common template (Ashburner, 2007). Grey matter maps were modulated, warped to Montreal Neurological Institute (MNI) space and smoothed with an isotropic Gaussian kernel of 8 mm full-width at half-maximum using the DARTEL toolbox ‘Normalize to MNI’ procedure. These smoothed, warped, modulated T1w images were used in independent ROI and whole-brain voxel-based morphometry (VBM) analyses.

### Neuroimaging analysis

#### ROI

To compare directly the relationship between ACC grey matter volume and belief updating in young and older adults, we used an ROI approach. We used a bilateral ACC atlas mask, obtained from the AAL toolbox (Tzourio-Mazoyer *et al.* 2002), to limit the number of statistical tests. Manual segmentation of this ACC mask into dorsal and ventral subregions was achieved using ITK-SNAP (Yushkevich *et al.* 2006), using established guidelines where the ventral portion was defined by drawing a line in the coronal plane at the tip of the corpus callosum (Killiany *et al.* 2000; Giuliani *et al.* 2011). We performed correlations between grey matter volume of the dorsal and ventral ACC and task measures in both age groups (two-tailed Pearson's correlations and partial Pearson's correlations with age, gender and total intracranial volume as covariates). We focused on the update bias (desirable update minus undesirable update) because this summary measure best captured the behavioural difference between age groups. The significance level for these correlations was *p* < 0.0125 (Bonferroni correction for four tests). Follow-up, *post-hoc* tests of dorsal ACC volume were performed using the components of the update bias (i.e. desirable update and undesirable update). For significant correlations among older adults, we tested whether these were significantly stronger in older than young adults using Fisher's *r* to *z* transformation.

#### VBM

We performed three exploratory VBM analyses. For all these analyses, no regions survived a statistical threshold of *p* < 0.05 after whole-brain peak-level family-wise error correction but, for completeness, we report the uncorrected results in Tables S5–S9. As no previous studies have reported the structural correlates of belief updating, for the first analysis (Tables S5–S7) we performed whole-brain VBM analyses across all participants. Regressors in this multiple regression model included update for desirable information, update for undesirable information, and age, gender and total intracranial volume (TIV, sum of grey matter, white matter and cerebrospinal fluid) as coviariates of no interest. In a second analysis (Table S8) we used update bias (desirable update minus undesirable update) and age, gender and TIV as covariates of no interest in a full factorial design to identify age interactions of the update bias (contrasts: update bias young > older and update bias older > young). The third analysis (Table S9) involved a conjunction analysis to identify regions that atrophied with age (contrast: young > older) and that correlated positively with undesirable update in young adults (contrast: young undesirable update > older undesirable update) to address the specific hypotheses that an enhanced update bias may emerge in older age due to age-related volume reduction of a region implicated in updating undesirable information (hence gender and TIV but not age were used as covariates of no interest in this model).

## Results

### Age comparison of updating beliefs

**Fig. 2.**
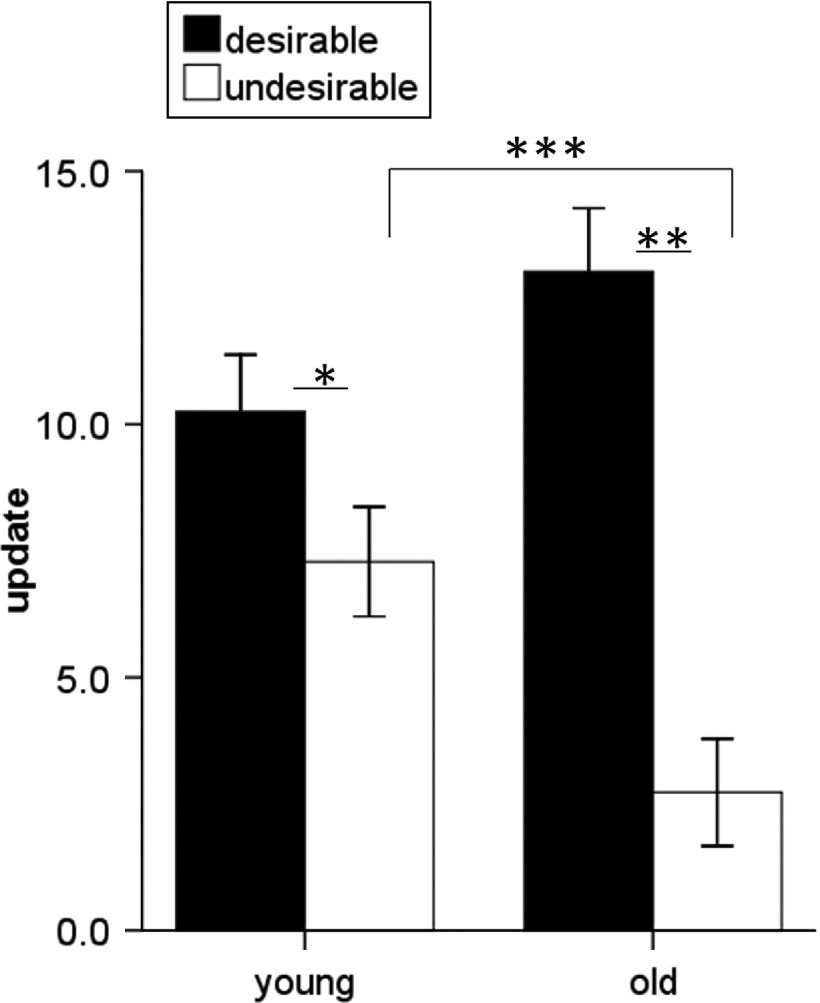
Age comparison of belief updating. Both young and older adults updated their beliefs (difference between first and second estimate) more when faced with desirable than undesirable information, but this update bias was larger in older adults due to reduced updating of undesirable information. *n* = 18 both groups. Bars ±1 s.e.m. * *p* < 0.05, ** *p* < 0.01, *** *p* < 0.0005.

The belief updating task (Fig. 1) was completed by 18 young and 18 older healthy adults. Our results show both an asymmetry between updating beliefs for desirable and undesirable information and a marked age-related difference in this update bias. Both young and older subjects displayed an update bias. In other words participants of all ages updated their beliefs more for desirable information than undesirable information (main effect of update valence: *F*_1,34_ = 58.29, *p* < 0.0005). This pattern was evident in 72% of younger adults and 94% of older adults. Importantly, older adults had a greater asymmetry in belief updating than younger adults, indicated by a significant interaction between update valence (desirable update/undesirable update) and age group (young/older) (*F*_1,34_ = 17.75, *p* < 0.0005). Follow-up tests showed that this interaction arose because older adults updated their beliefs for undesirable information even less than younger adults (*t*_34_ = 3.01, *p* = 0.005), whereas both age groups updated their beliefs for desirable information to a similar extent (*t*_34_ = 1.65, *p* = 0.109), resulting in a greater update bias among older adults (Fig. 2).

We measured several other variables, and to determine whether these variables contributed to the age-related difference in update bias, we included them all as covariates in the analysis of update bias. First, reaction times did not differ between age groups (see Supplementary Results; Table S2). Second, the number of trials classified as desirable and undesirable differed between age groups (number of desirable trials: young: mean = 18, s.d. = 5.52; older: mean = 13, s.d. = 5.10, *t*_34_ = −2.92, *p* = 0.006; number of undesirable trials: young: mean = 25, s.d. = 5.80; older: mean = 30, s.d. = 4.53, *t*_34_ = 2.88, *p* = 0.007). Third, older adults had a greater tendency than young adults to initially underestimate the likelihood of negative events, indicated by a more negative first estimation error (young: mean = −4.71, s.d. = 6.26; older: mean = −12.24, s.d. = 6.29; first estimation error young *versus* older: *t*_34_ = 3.60, *p* = 0.001; Table S3).

Fourth, we tested participants' recollection for the average probabilities for each event presented during the task (Table S4). Older adults made more memory errors (calculated as the absolute difference between the average probability for each event and the participants' recollection of that number) than younger adults (main effect of age: *F*_1,35_ = 9.65, *p* = 0.004). However, memory errors were similar for trials when desirable and undesirable information were encountered (main effect of valence: *F*_1,34_ = 1.44, *p* = 0.238; valence × age interaction: *F*_1,34_ = 0.52, *p* = 0.477).

Fifth, an analysis of subjective rating scales revealed that all participants rated trials where they received desirable information as more vivid, more familiar and indicated greater past experience of these events compared to trials where they received undesirable information (main effect of valence, vivid: *F*_1,34_ = 86.98; familiar: *F*_1,34_ = 44.74; experience: *F*_1,34_ = 87.84; all *p* < 0.0005). All participants rated trials where they received undesirable information as more arousing and more negative than trials where they received desirable information (main effect of valence, arousal: *F*_1,34_ = 16.55; negative: *F*_1,34_ = 20.12; all *p* < 0.0005). An age-related difference was only present for ratings of arousal (main effect of age: *F*_1,34_ = 13.89, *p* = 0.001; all other main effects of age *p* > 0.1) and familiarity (valence × age interaction: *F*_1,34_ = 7.49, *p* = 0.010; all other valence × age interactions *p* > 0.1) (see Supplementary Results). In fact, older adults rated all events as more emotionally arousing than younger adults. This would suggest that the greater update bias in older adults was not due to participants being less engaged in the task or finding the stimuli less relevant than younger adults did. Familiarity ratings indicated how familiar participants were with each event regardless of their personal experience. Here we found a trend towards older adults rating events in which they received desirable information as more familiar than younger adults (*t*_34_ = 1.82, *p* = 0.078) and no age group difference for familiarity for events in which they received undesirable information (*t*_34_ = 0.08, *p* = 0.939). Overall, the subjective ratings analyses suggest that the task events were just as salient for older as for young adults and the relative lack of interactions with age group make it highly unlikely that these variables accounted for the age-related difference in the update bias.

Finally, we used the LOT-R self-rating personality scale as an independent measure of trait optimism and found that older adults had higher trait optimism scores (mean = 20.17, s.d. = 2.88) than young adults (mean = 16.56, s.d. = 3.26) (young *versus* older: *t*_34_ = 3.53, *p* = 0.001).

To fully account for the differences detailed here, we added the following measures as covariates to the analysis of the update bias: IQ, LOT-R score, first estimation error, difference measures (desirable minus undesirable) of all five subjective rating scales (vividness, familiarity, experience, emotional arousal, negativity), difference measures (desirable minus undesirable) of reaction times, average memory errors and number of desirable and undesirable trials. Importantly, the significantly greater update bias in older adults persisted even when all these factors were included as covariates (ANCOVA update, valence × age interaction: *F*_1,21_ = 8.48, *p* = 0.008).

### ACC volume and belief updating

**Fig. 3.**
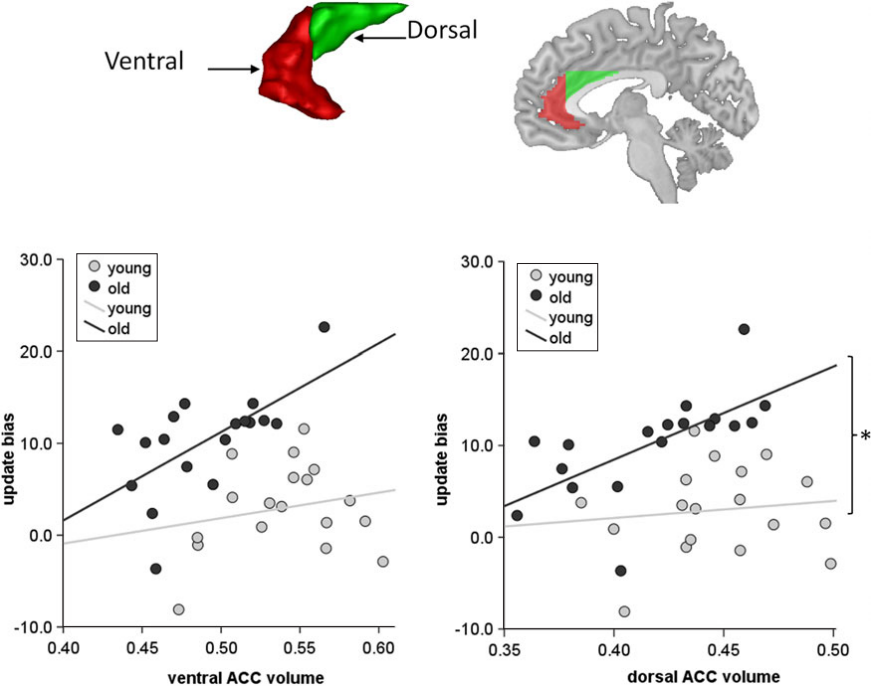
Region of interest (ROI) analysis of the anterior cingulate cortex (ACC). Scatter plots show higher volume of the dorsal ACC (green mask) and ventral ACC (red mask) in older adults correlated with higher update bias (desirable update minus undesirable update) in older adults. No significant correlations were observed in young adults. *n* = 18 for each age group. * Fisher's *r* to *z* transformation comparing correlation strengths *p* < 0.05.

We performed a structural neuroimaging analysis in relation to our *a priori* region of interest, the ACC. Functional activity in this region has been associated with a positivity effect in older age whereas the relationship between structural volume and positivity in older age is unknown. We therefore parcellated an anatomically defined bilateral mask of this region to obtain grey matter volume of dorsal and ventral ACC subregions and examined the correlation with updating beliefs and age group differences. We found that the volume of both the dorsal and ventral ACC regions correlated positively with the update bias in older adults whereas neither region correlated with update bias in younger adults (Table 1; see also Table S10). The dorsal ACC correlation in older adults was significantly greater than in young adults (Fisher's *r* to *z, z* = −1.78, *p* = 0.036), in contrast to the non-significant age group difference for the ventral ACC (*z* = −1.42, *p* = 0.078) (Fig. 3). There was no interaction with region (dorsal ACC *versus* ventral ACC correlation with update bias) for either older adults (Fisher's *r* to *z, z* = 0.11, *p* = 0.456) or young adults (Fisher's *r* to *z, z* = −0.26, *p* = 0.397). Greater dorsal ACC volume also correlated with a higher desirable update in older adults (Fig. S1) although not after controlling for age, gender and TIV (Table S11). In summary, the strongest correlation we identified after controlling for other variables was between the update bias and dorsal ACC volume among older adults.

**Table 1.** Correlation coefficients (Pearson's correlations) for the correlation between dorsal and ventral subregions of anterior cingulate cortex (ACC) grey matter volume and update bias (desirable update minus undesirable update)

	Older adults		Young adults	
	*r*	*p*	*r*	*p*
Dorsal ACC	0.649, 0.526	0.004*, 0.044	0.122, 0.057	0.631, 0.841
Ventral ACC	0.626, 0.504	0.005*, 0.056	0.213, 0.262	0.395, 0.345

*r* = Pearson's correlation coefficient (first value in cell) and partial Pearson's correlation coefficient controlling for age, gender and total intracranial volume (second value in the same cell of the table). *p* = corresponding significance value, *p < 0.0125.

The overall volume of both dorsal (young: mean = 0.45, s.d. = 0.032; older: mean = 0.42, s.d. = 0.036) and ventral ACC (young: mean = 0.54, s.d. = 0.037; older: mean = 0.49, s.d. = 0.036) was reduced in older age (dorsal: *t*_34_ = −2.55, *p* = 0.015; ventral: *t*_34_ = −4.08, *p* < 0.0005). To further assess whether the association between volumes of ACC subregions and update bias differed between age groups because of the range of volume values, we formed age groups matched for volume. We excluded young adults with dorsal ACC volumes higher than the maximum volume in older adults (*n* = 3) and excluded older adults with lower dorsal ACC volumes than the minimum volume in young adults (*n* = 4). This did not change the pattern of results, whereby a strong correlation remained in older adults (*r* = 0.72, *p* = 0.004) and there remained no correlation among young adults (*r* = 0.34, *p* = 0.209). Using the same approach for the ventral ACC, we excluded young adults with higher ventral ACC volumes than the maximum volume in older adults (*n* = 3) and older adults with lower ventral ACC volumes than the minimum volume in young adults (*n* = 7). Here, despite the small sample size, the significant correlation persisted in older adults (*r* = 0.72, *p* = 0.013). Additionally, an almost significant correlation emerged in young adults (*r* = 0.51, *p* = 0.051). Overall, these results suggest that individual differences in dorsal ACC volume relate to the update bias in older adults compared to young adults despite age-related volume differences.

Overall, our results suggest that a greater relative volume of the ACC (that is a greater volume among older adults but not a greater volume independent of age) is coupled to a greater update bias in older age. Another potential explanation for the greater update bias in older age is that the greater failure of older adults to update undesirable information occurs as a consequence of age-related brain atrophy. To assess this we performed an exploratory whole-brain VBM conjunction analysis (see Method) that identified clusters in the superior and middle temporal lobe, superior frontal gyrus and cerebellum, indicating regions that were both reduced in volume in older adults and correlated with undesirable update more in younger than older adults, yet none of these regions survived whole-brain correction for multiple comparisons (see Table S9).

## Discussion

We provide evidence that older adults are less likely than younger adults to change their beliefs when faced with undesirable information about their future, resulting in a greater update bias in older age. This greater asymmetry in belief updating was present despite older adults experiencing greater subjective feelings of emotional arousal for all task events. This update bias was also independent of non-age-related valence differences in subjective ratings of the task stimuli, including the sense of personal experience, vividness, familiarity, emotional arousal and negativity, whereby all these measures were included as covariates in our analyses. The observed age difference in update bias was not a consequence of a general memory deficit because there was no age difference between memory recollection for the average probabilities of desirable and undesirable events and, importantly, the interaction between age and the update bias persisted when overall poorer memory performance in older age was accounted for in our analysis. Finally, the greater update bias in older age persisted after controlling for the enhanced tendency by older adults to initially underestimate the likelihood of occurrence of negative events relative to the average statistics and after controlling for optimistic trait personality scores.

Our study provides preliminary evidence that a greater asymmetry in belief updating in older adults is tightly coupled to the relative volume of the ACC. Previous studies report a crucial role of the ACC as a cognitive-emotional interface, although this evidence has been derived from functional neuroimaging studies (for reviews, see Bush *et al.* 2000; Ochsner & Gross, 2005). We acknowledge that the relationship between structural and functional changes in the ageing brain is likely to be complex, yet it is notable that our structural findings are broadly in line with studies reporting a link between greater functional ACC activity and more emotional regulation in older age (Brassen *et al.* 2011, 2012; Samanez-Larkin & Carstensen, 2011). We show that the volume of the dorsal subregion of ACC was more strongly associated with the update bias in older than young age. Moreover, even after matching for the range of volume of ACC subregions across age groups, no correlation emerged in younger adults between update bias and volume of the dorsal subregion, suggesting this relationship was exclusive to older adults. The volume of the dorsal as opposed to the ventral ACC, often dubbed ‘cognitive’ and affective’ regions respectively (Devinsky *et al.* 1995; Bush *et al.* 2000), has in fact been linked to greater cognitive reappraisal strategies in healthy adults (Giuliani *et al.* 2011), and structural volume of this subregion is reduced in patients with depression, where emotional dysregulation and pessimism are highly characteristic (Vasic *et al.* 2008). However, functional activity within the rostral ACC has also been linked to an optimistic bias in younger adults (Blair *et al.* 2013) and to greater emotional stability in older adults (Brassen *et al.* 2011, 2012). Therefore, it remains a challenge for future studies to clarify the dissociation between ACC subregions and positivity effects in older age.

Intriguingly, we found a positive correlation such that older adults with greater dorsal ACC volume had a larger update bias. This was despite an overall age-related decline in ACC volume, emphasizing that the link here is between relatively greater volume among older adults. We speculate that this could indicate that older adults, when integrating beliefs with experience, rely more on this region than younger adults. More generally, this interpretation is in keeping with the socio-emotional selectivity theory, in which a positivity bias may be viewed as an enhanced phenomenon in older age (Samanez-Larkin & Carstensen, 2011; Nashiro *et al.* 2012) rather than a ‘side-effect’ of age-related structural decline as has been proposed, for example, by the ageing brain model (Cacioppo *et al.* 2011).

To explore the latter alternative explanation, we also performed a whole-brain VBM analysis to determine whether age-related decline of any other brain regions was associated with a failure of updating undesirable beliefs in older age, yet we did not find strong evidence for this. We were alert to the potential role of other brain structures in belief updating that our ROI approach would not account for, also motivating additional whole-brain VBM analyses. We acknowledge that the overall lack of significant VBM findings may have been due to our small sample size and we do not exclude the possibility that additional mechanisms and brain structures may well contribute to the enhanced update bias in older age. Sociocultural factors specific to our cohorts might account for the differences we found in optimism between our young and older participants. These are necessarily more difficult to determine with our cross-sectional design, but we note that large-scale longitudinal studies have demonstrated fewer negative emotions in older age (Charles *et al.* 2001; Carstensen *et al.* 2011).

As in the study by Sharot *et al.* (2011), we only examined negative events because an update bias here may have an adverse impact on health-protective behaviours (Weinstein & Klein, 1995). An optimistic update bias for positive events has in fact been observed in other studies (Eil & Rao, 2011; Korn *et al.* 2012). Further study using positive events would be required to test whether this remains the case in older age.

In summary, we show that healthy older adults display an enhanced update bias compared to younger adults, an effect that correlates with grey matter volume of the ACC. Our findings relate to a small sample of healthy older individuals with a high IQ. A logical extension of our study would be to examine how IQ and age-associated pathologies impact on updating beliefs. One important condition would be later-life depression (Alexopoulos, 2005), where our results have particular relevance given the link between ACC structure and the update bias that we highlight, in addition to reports for an association between structural ACC abnormalities and clinical depression (Vasic *et al.* 2008).

In our ageing society, we provide a timely demonstration of an age-related enhancement of the update bias that has important implications for healthy ageing. Optimism has been related to better physical health (Scheier *et al.* 1999; Diener & Chan, 2011; Kim *et al.* 2011), indicating that an enhanced optimistic update bias may convey adaptive benefits in older age. For example, an optimistic processing bias may allow older adults to maintain the same level of happiness and well-being when faced with the many health and personal challenges that older age entails, such as death of loved ones and financial difficulties. However, a broad implication of our findings is that the presence of such a bias may skew economic, personal and health-related decisions. For example, older adults may make inappropriate insurance purchases based on a false optimism in relation to their future. Indeed, an age-related increase in risky financial decision making has been described (Samanez-Larkin *et al.* 2010, 2011), which could be linked to less updating after losses (i.e. after worse than expected outcomes) but more equivalent updating after gains (i.e. after better than expected outcomes). Thus, our findings may help to further elucidate ‘real-world’ decision-making processes in healthy ageing.

## Supplementary Material

Supplementary Material Supplementary information supplied by authors.

Supplementary Material Supplementary information supplied by authors.

## Supplementary material

For supplementary material accompanying this paper, visit http://dx.doi.org/10.1017/S0033291713002602.
