# Supplementary figures and images for: Optimistic update bias increases in older age
Source: Psychol Med. 2013 Nov 4;44(9):2003–12. doi: 10.1017/S0033291713002602 (PMC4035755; doi:10.1017/S0033291713002602)

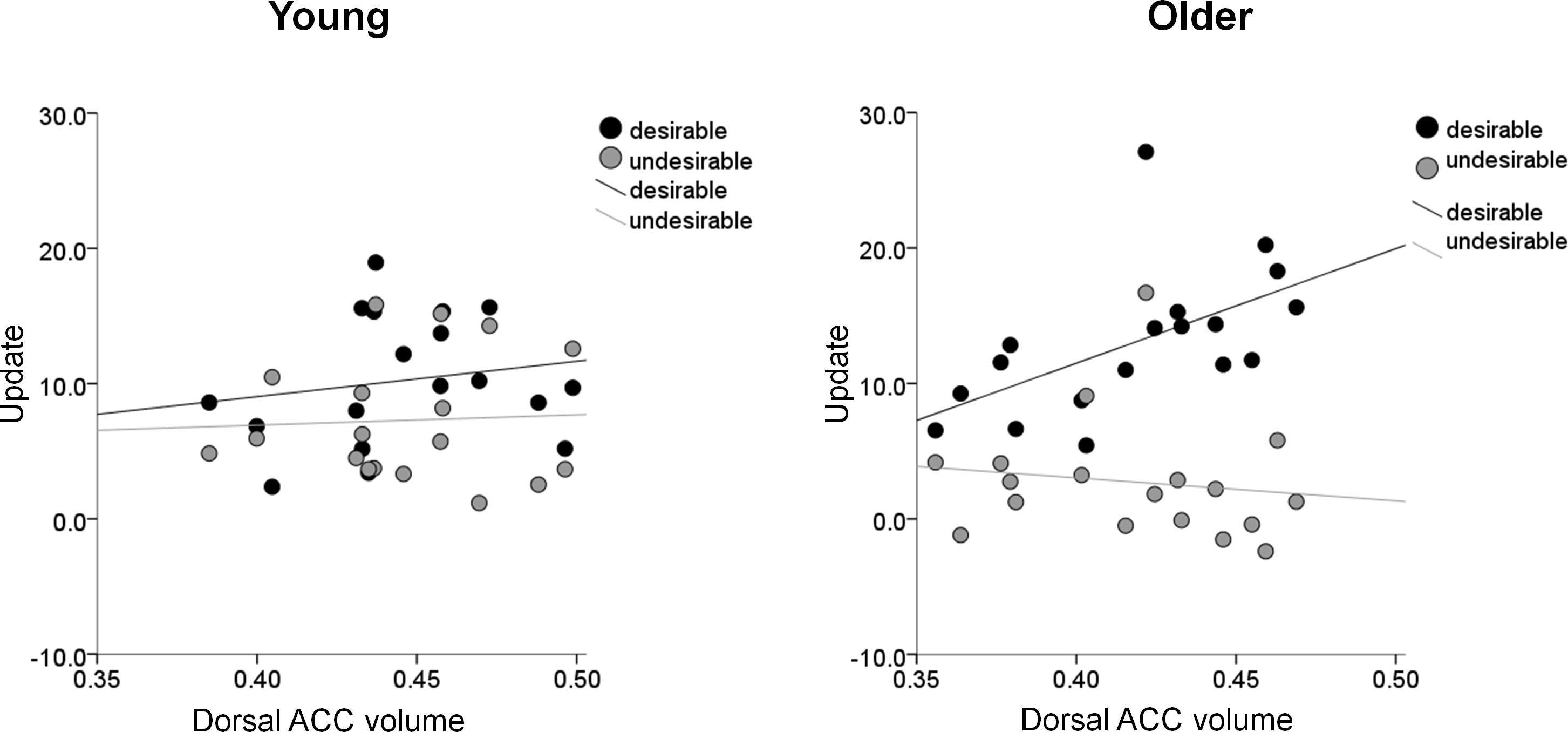

Supplement: Supplementary Material — Supplementary information supplied by authors. [file S0033291713002602sup001.tif]
