# Supplementary material for: Optimistic update bias increases in older age
Source: Psychol Med. 2013 Nov 4;44(9):2003–12. doi: 10.1017/S0033291713002602 (PMC4035755; doi:10.1017/S0033291713002602)
Supplement: Supplementary Material — Supplementary information supplied by authors. [file S0033291713002602sup002.doc]

**Supplementary Material for: Optimistic update bias increases in older age**

Rumana Chowdhury, Tali Sharot, Thomas Wolfe, Emrah Düzel and Raymond J Dolan

**Supplementary Method**

**Participants:** To ensure participants were healthy, they were initially screened by telephone and excluded if they had any of the following: current or past history of neurological, psychiatric or endocrinological disorders (including diabetes mellitus and thyroid dysfunction), major visual or hearing impairment, history of drug addiction and current illicit drug use. To control for vascular risk factors, individuals known to have had a stroke or transient ischemic attack, myocardial infarction or require more than one anti-hypertensive medication were not eligible for participation. Participants with any contraindications to MRI scanning were not eligible for participation.

**Cognitive screening:** All participants had a Mini-Mental State Examination (MMSE) score ≥28. Since the MMSE alone is not a sensitive marker of pathology, in older adults we administered additional standardised neuropsychological tests to screen for deficits in declarative memory (Rey Auditory Verbal Learning Test, RAVLT immediate and delayed recall), visuo-motor speed (Digit Symbol Substitution Test, DSST), attention and set-shifting (Trail-making A & B). We excluded participants who scored >1SD outside the age-related norms for the cognitive tests to ensure older adults had intact global cognitive function. This resulted in the exclusion of two participants based on their RAVLT delayed free recall score, where low scores may be an early indicator of pathology (1). For the remaining older participants, mean (SD) cognitive scores were as follows: RAVLT immediate recall 55.50 (6.33), RAVLT delayed recall 11.33 (2.23), DSST score 56.67 (8.22), Trail-Making A time 29.55 sec (6.19), Trail-Making B time 58.51 sec (20.04).

**Mood screening**: We measured depressive symptoms in all participants using the Beck Depression Inventory (2). BDI scores <11 are considered within the normal range, therefore we excluded one young and three older participants with BDI scores > 10 (note one of these older adults was also excluded on the basis of their low RAVLT score as described above).

**Additional details about the belief updating task:** As previously described by Sharot et al (2011), the average probability of each event occurring to a person living in the same socio-cultural environment was determined using online resources (Office for National Statistics, Eurostat, Pubmed) and additionally in this study, using a small number of events from a previously validated set of events likely to occur to the general population (3). Very rare and very common events were not included thus all event probabilities lay between 10% and 70%. Participants were told the range of probabilities was between 3% and 77% to ensure the ranges of possible overestimation and underestimation were equal. As in the study by Sharot et al (2011), we only examined negative events since an update bias here may have an adverse impact on health-protective behaviours (4).

Trials in which the estimation error was zero or participants did not respond were discarded. On average, both young and old adults completed most trials (mean 44.8, SD 0.73 and mean 44.1, SD 1.48 respectively).

**Anatomical MRI acquisition:** A high resolution structural MRI data set was acquired on a 3.0T Trio MRI scanner (Siemens) using a 32-channel head coil. Two sets of a multiparameter map protocol at 0.8mm isotropic resolution were acquired for each subject and averaged into a single data set to improve the signal-to-noise ratio. This 3D multi-echo fast low angle shot (FLASH) sequence was used to acquire T1-weighted images (TE 2.2-9.85ms, TR 23.7ms, FA 28 degrees) (5). B1 mapping (TE 39.38 and 19.69ms, TR 500ms, FA 270:10-180 degrees, 4mm^3^ isotropic resolution) was acquired to correct the T1 maps for inhomogeneities in the transmit radiofrequency field (6). A double-echo FLASH sequence (TE1 10ms, TE2 12.46ms, 3 x 3 x 2 mm resolution and 1mm gap) was used to measure local field inhomogeneities and correct for the image distortions in the B1 mapping data.

**Supplementary results**

**Reaction times:** Reaction times were analysed using a repeated measures ANOVA with session (first / second) and valence (desirable / undesirable) as within-subjects factors and age-group (young / old) as the between-subjects factor. This analysis showed that reaction times did not differ between age-groups (session*valence*age interaction: F(1,34) = 2.73, p = .108). All participants had slower reaction times when entering their first estimate compared to the second (main effect of session: F(1,34) = 16.14, p<0.0005). For the first session alone all participants were slower on undesirable compared to desirable trials (session*valence interaction: F(1,34) = 4.70, p = .037). We found a session (first / second) by valence (desirable/undesirable) interaction (F(1,34) = 4.70, p = .037). This was due to faster responses for desirable trials compared to undesirable trials on the first session (t(35) = -2.13, p = .040), whereas there was no difference between desirable and undesirable trials on the 2^nd^ session t(35) = 0.51, p = .617) (**Table S2**).

**Subjective Rating Scales:** There were no age differences in the sense of personal experience (main effect if age: F(1,34) = 0.02, p = .891) or how vivid (main effect if age: F(1,34) = 2.75, p = .106), familiar (main effect if age: F(1,34) = 0.05, p = .0826) or negative (main effect if age: F(1,34) = .01, p = .931) participants’ rated the adverse task events.

There were no age-related valence differences in the sense of personal experience (valence*age F(1,34) = 1.76, p = .194) or how vivid (valence*age F(,134) = 2.53, p = .121), arousing (valence*age F(1,34) = 0.75, p = .394) or negative (valence*age F(1,34) = 0.83, p = .367) participants’ rated the adverse task events.

Figure S1. Scatter plots showing associations between dorsal anterior cingulate cortex (ACC) volume and desirable and undesirable update in young and older adults. See also Table S10.

Functional activity of dorsal ACC has also been linked to signalling surprise, when outcomes do not match expectations (7) (8). Given that the frequency of desirable trials in our task was even less amongst older adults than young adults, desirable trials may be considered as more surprising for the older cohort. This may be an additional explanation as to why dorsal ACC volume was more strongly associated with desirable update in older but not young adults. However we acknowledge this interpretation is speculation as there may not be a direct mapping between functional activity within ACC and structural volume of ACC.


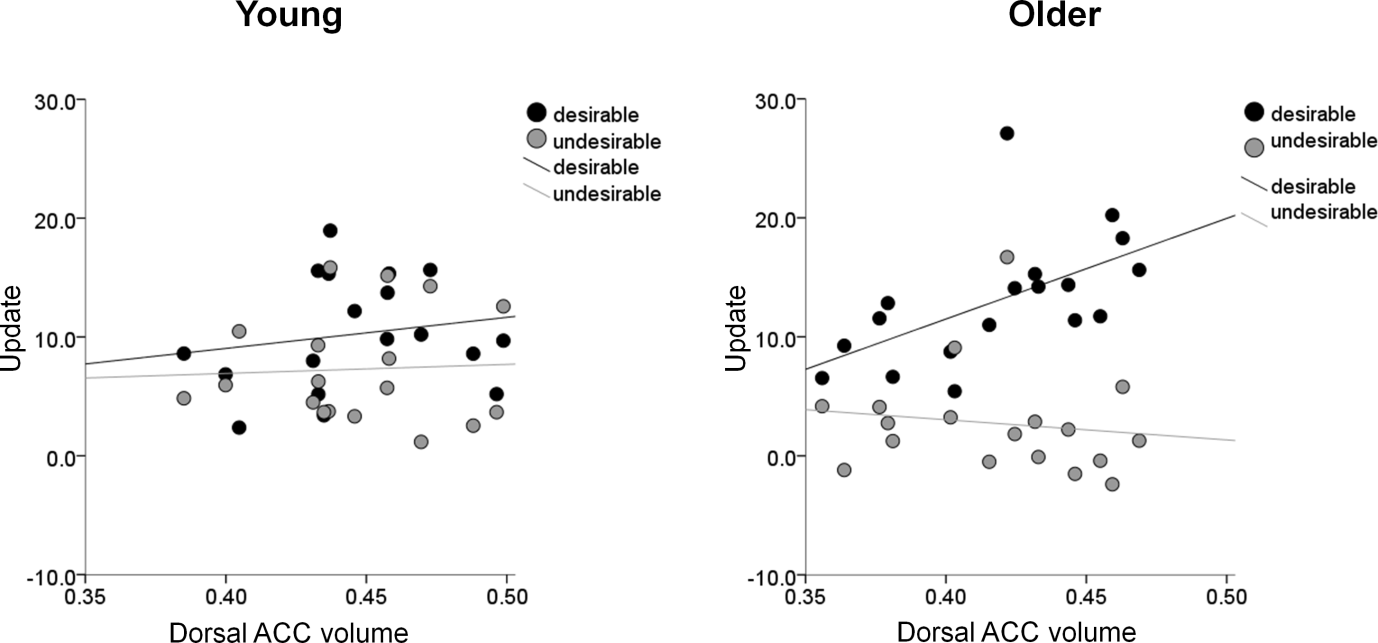


**Table S1. Sample characteristics** (18 participants per group). Mean (SD) or median (range). Independent t-tests for parametric variables, Mann-Whitney U test for non-parametric variables. IQ estimated using the National Adult Reading Test; MMSE: Mini-Mental State Examination; BDI: Beck Depression Inventory

|  | **Young** | **Old** | **t / Z** | **p** |
| --- | --- | --- | --- | --- |
| Age (yrs) | 22. 22 (2.29) | 66.00 (5.62) | 30.63 | <0.0005 |
| Gender (M:F) | 8:10 | 7:11 | 0.33 | .791 |
| Yrs education | 17.11 (1.18) | 16.83 (2.01) | 0.51 | .616 |
| IQ | 110.72 (7.88) | 123.83 (6.19) | 5.55 | <0.0005 |
| MMSE | 30 (29 – 30) | 30 (29 – 30) | 0.81 | .613 |
| BDI | 3.06 (2.10) | 4.00 (2.30) | 1.26 | .216 |

**Table S2. Reaction times.** Mean reaction times in seconds, SD in parentheses. Group average indicates mean reaction time average across young (n = 18) and older (n = 18) participants.

|  | **Young** | **Older** | **Group average** |
| --- | --- | --- | --- |
| **First session** |  |  |  |
| Desirable trials | 3.60 (0.83) | 4.17 (1.19) | 3.89 (1.05) |
| Undesirable trials | 3.94 (0.78) | 4.28 (1.62) | 4.11 (1.27) |
| **Second session** |  |  |  |
| Desirable trials | 3.94 (0.57) | 3.50 (0.78) | 3.49 (0.67) |
| Undesirable trials | 3.37 (0.62) | 3.55 (0.88) | 3.46 (0.75) |

**Table S3: Initial estimations by young and older adults for each event**

Older adults had a greater tendency than young adults to initially underestimate the likelihood of negative events (more negative first estimation error). We included this measure as a covariate in our analysis to ensure this was not a confounding variable. However, this more negative first estimation error in older adults may also indicate that older adults had a more ‘optimistic’ outlook at the outset of the task. Therefore we provide the first estimation for all events in the table below (events ranked in order of the absolute initial estimation difference between age-groups). For the majority of events (82%), older adults’ initial estimation was lower than younger adults. No clear pattern emerged regarding the categorisation of these events. For example older adults showed a greater initial underestimation both for events that may be perceived as under the control of the individual (e.g. incurring debts, late for a meeting), those which may be perceived as random (e.g. accidents) and included both social (e.g. disagreement with friend) and financial (e.g. bounce cheque) events.

|  | **young** |  | **old** |  |  |
| --- | --- | --- | --- | --- | --- |
|  | **mean** | **SD** | **mean** | **SD** | **difference** |
| **First estimate old < young** |  |  |  |  |  |
| more than £30000 debts | 29.50 | 30.03 | 2.71 | 0.85 | 26.79 |
| more than 15 minutes late for an important meeting | 49.67 | 19.15 | 23.72 | 25.20 | 25.94 |
| accidentally break something at a friends' house | 48.11 | 18.43 | 23.28 | 15.37 | 24.83 |
| sport related accident | 34.17 | 19.02 | 12.94 | 14.1 | 21.22 |
| public transport delay causing you to be late | 74.28 | 8.46 | 53.06 | 20.51 | 21.22 |
| short-changed in a shop | 50.33 | 22.01 | 33.22 | 25.27 | 17.11 |
| burn something you are cooking | 60.44 | 22.63 | 43.94 | 28.68 | 16.50 |
| miss a flight | 26.06 | 22.06 | 9.94 | 7.74 | 16.11 |
| lose your house keys | 49.00 | 19.97 | 33.33 | 24.43 | 15.67 |
| being cheated by husband/wife/partner | 24.56 | 16.78 | 8.94 | 12.18 | 15.61 |
| victim of mugging | 29.33 | 15.3 | 15.72 | 7.74 | 13.61 |
| your cheque/payment bounces | 24.11 | 13.75 | 11.06 | 16.36 | 13.06 |
| skin burn | 49.83 | 23.17 | 37.12 | 26.62 | 12.72 |
| victim of violence by acquaintance | 17.56 | 13.17 | 6.25 | 6.82 | 11.31 |
| theft from vehicle | 28.22 | 21.58 | 18.61 | 18.52 | 9.61 |
| serious disagreement with a good friend | 31.72 | 16.55 | 22.76 | 20.26 | 8.96 |
| receive unwanted call from telemarketer | 71.28 | 10.68 | 62.61 | 25.35 | 8.67 |
| passenger in a car accident | 21.67 | 7.85 | 13.06 | 8.44 | 8.61 |
| heating system in your house breaks down | 45.22 | 18.02 | 36.65 | 24.79 | 8.58 |
| stung by a bee | 27.78 | 17.49 | 19.89 | 17.88 | 7.89 |
| victim of violence at home | 13.56 | 12.58 | 6.29 | 6.69 | 7.26 |
| car/bicycle stolen | 34.83 | 15.41 | 28.39 | 22.21 | 6.44 |
| have a serious family argument | 35.50 | 22.79 | 29.06 | 28.01 | 6.44 |
| get a parking or speeding ticket | 31.78 | 19.64 | 26.41 | 27.11 | 5.37 |
| theft from your person | 32.17 | 17.18 | 26.94 | 18.84 | 5.22 |
| domestic burglary | 30.61 | 14.34 | 25.5 | 16.62 | 5.11 |
| spill substance (e.g. red wine) and stain carpet | 56.72 | 23.76 | 51.89 | 22.44 | 4.83 |
| victim of violence by stranger | 16 | 8.75 | 11.5 | 10.15 | 4.50 |
| computer crash with loss of important data | 36.53 | 14.38 | 32.35 | 21.66 | 4.18 |
| victim of violence with need to go to A&E | 14.22 | 8.48 | 10.28 | 4.61 | 3.94 |
| car/bicycle vandalised | 31.22 | 15.8 | 27.33 | 22.32 | 3.89 |
| household accident | 45.39 | 20.98 | 42.06 | 23.38 | 3.33 |
| shouted at by a stranger | 47.39 | 20.07 | 44.12 | 22.65 | 3.27 |
| being convicted of crime | 8.76 | 6.51 | 5.59 | 4.57 | 3.18 |
| having fleas/lice | 15.12 | 9.46 | 12 | 16.02 | 3.12 |
| fraud when buying something on the internet | 30.89 | 19.11 | 28.29 | 18.86 | 2.59 |
| holiday cancelled due to natural disaster | 19.72 | 12.32 | 18.72 | 10.5 | 1.00 |
| **First estimate young < old** |  |  |  |  |  |
| insect infestation (e.g. ants) in your home | 35.89 | 24.13 | 50.56 | 23.36 | 14.67 |
| identity fraud | 18.39 | 11.74 | 30.47 | 18.13 | 12.08 |
| card fraud | 28.39 | 13.98 | 33.78 | 19.82 | 5.39 |
| roof leak | 26.06 | 15.87 | 30 | 24.74 | 3.94 |
| severe injury due to accident (traffic or house) | 19.61 | 11.72 | 21.89 | 17.23 | 2.28 |
| find mouse in your house | 40.61 | 27.71 | 41.94 | 28.92 | 1.33 |
| witness a traumatising accident | 21.94 | 14.92 | 22.94 | 14.47 | 1.00 |
| house vandalised | 16.22 | 8.26 | 17.06 | 13.14 | 0.84 |

**Table S4. Subjective ratings and memory performance.** Subjective ratings are scores measured using a Likert scale ranging from 1 (not at all) to 6 (very) for all 45 task events. Memory errors are the absolute difference between the actual probability presented for each adverse event and the participants’ recollection of those actual probabilities. Scores are mean, SD in parentheses.

|  | **Vivid** | **Familiar** | **Experience** | **Arousal** | **Negative** | **Memory errors** |
| --- | --- | --- | --- | --- | --- | --- |
| **Group average (n = 36)** | |  |  |  |  |  |
| Desirable | 4.38 (0.81) | 4.08 (0.81) | 2.64 (0.55) | 3.62 (0.81) | 3.72 (0.88) | 9.79 (4.19) |
| Undesirable | 3.52 (0.83) | 3.48 (0.93) | 1.82 (0.29) | 3.95 (0.79) | 4.09 (0.77) | 10.67 (3.38) |
| **Young (n =18)** | |  |  |  |  |  |
| Desirable | 4.03 (0.52) | 3.84 (0.78) | 2.50 (0.51) | 3.30 (0.71) | 3.71 (0.49) | 8.08 (2.98) |
| Undesirable | 3.32 (0.66) | 3.49 (0.86) | 1.79 (0.30) | 3.46 (0.78) | 4.15 (0.59) | 9.49 (3.18) |
| **Older (n = 18)** | |  |  |  |  |  |
| Desirable | 4.73 (0.91) | 4.32 (0.78) | 2.79 (0.56) | 3.95 (0.79) | 3.74 (0.12) | 11.51 (4.59) |
| Undesirable | 3.52 (0.83) | 3.46 (0.10) | 1.84 (2.87) | 4.34 (0.58) | 4.03 (0.93) | 11.86 (3.24) |

**Table S5. Positive correlation with desirable update across all participants. Uncorrected threshold p < 0.001, > 10 voxels. No regions correlated negatively with desirable update.**

| **No. voxels** | **T** | **Z** | **x** | **y** | **z** | **L/R** | **region** |
| --- | --- | --- | --- | --- | --- | --- | --- |
| 3219 | 4.99 | 4.23 | -16 | -43 | -47 | L | cerebellum |
| 1294 | 4.88 | 4.16 | 36 | -35 | 62 | R | postcentral |
| 1706 | 4.48 | 3.89 | 49 | -14 | -30 | R | inferior temporal |
| 293 | 4.27 | 3.74 | -38 | -30 | -22 | L | fusiform |
| 1146 | 4.24 | 3.72 | -62 | -18 | 26 | L | postcentral |
| 398 | 4.06 | 3.60 | -49 | -13 | -28 | L | inferior temporal |
| 261 | 4.04 | 3.58 | 41 | -63 | 12 | R | mid temporal |
| 449 | 3.97 | 3.53 | 10 | 50 | 12 | R | anterior cingulate |
| 487 | 3.92 | 3.49 | -50 | 8 | 32 | L | inferior frontal gyrus |
| 1071 | 3.90 | 3.48 | 22 | 7 | 53 | R | superior frontal |
| 587 | 3.87 | 3.46 | -30 | -3 | -22 | L | hippocampus |
| 320 | 3.81 | 3.41 | -34 | 2 | 2 | L | insula |
| 83 | 3.75 | 3.37 | 23 | 46 | 28 | R | mid frontal |
| 150 | 3.74 | 3.36 | -6 | 1 | 70 | L | supplementary motor area |
| 89 | 3.69 | 3.32 | 42 | -25 | 47 | R | postcentral |
| 37 | 3.61 | 3.27 | -20 | -82 | 26 | L | superior occipital |
| 100 | 3.60 | 3.25 | 2 | -13 | 31 | R | mid cingulum |
| 28 | 3.57 | 3.23 | 29 | -78 | 22 | R | superior occipital |
| 39 | 3.54 | 3.21 | 29 | -51 | -53 | R | cerebellum |
| 29 | 3.53 | 3.21 | 28 | 17 | 42 | R | mid frontal |
| 18 | 3.53 | 3.20 | 56 | -33 | -21 | R | inferior temporal |
| 10 | 3.50 | 3.18 | 56 | -54 | 19 | R | mid temporal |
| 14 | 3.48 | 3.16 | 46 | -50 | 18 | R | mid temporal |
| 12 | 3.45 | 3.14 | 44 | 22 | 9 | R | interior frontal gyrus |
| 17 | 3.43 | 3.13 | -6 | 16 | 62 | L | supplementary motor area |

**Table S6. Correlations with undesirable update across all participants. Uncorrected threshold p < 0.001, > 10 voxels.**

| **No. voxels** | **T** | **Z** | **x** | **y** | **z** | **L/R** | **region** |
| --- | --- | --- | --- | --- | --- | --- | --- |
| **Undesirable negative** | | |  |  |  |  |  |
| 368 | 4.00 | 3.55 | 58 | -12 | 30 | R | postcentral |
| **Undesirable positive** | | |  |  |  |  |  |
| 118 | 3.96 | 3.52 | -16 | -80 | 22 | L | superior occipital |
| 175 | 3.74 | 3.36 | -7 | -83 | -23 | L | cerebellum |

**Table S7. Correlation with update bias (desirable update > undesirable update) across all participants.**  Uncorrected threshold p < 0.001, > 10 voxels.

| **No. voxels** | **T** | **Z** | **x** | **y** | **z** | **L/R** | **region** |
| --- | --- | --- | --- | --- | --- | --- | --- |
| 339 | 4.34 | 3.79 | -17 | -80 | 23 | L | superior occipital |
| 899 | 4.27 | 3.75 | 59 | -13 | 29 | R | supramarginal |
| 871 | 3.97 | 3.53 | 37 | -29 | 60 | R | postcentral |
| 57 | 3.85 | 3.44 | -38 | -29 | -22 | L | inferior temporal |
| 52 | 3.77 | 3.39 | 34 | -58 | -53 | R | cerebellum |
| 88 | 3.72 | 3.35 | -14 | -58 | -53 | L | cerebellum |
| 74 | 3.71 | 3.34 | -29 | -1 | -18 | L | amygdala |
| 223 | 3.67 | 3.31 | -13 | -4 | 69 | L | supplementary motor area |
| 59 | 3.60 | 3.26 | -18 | -42 | -46 | L | cerebellum |
| 96 | 3.58 | 3.24 | 23 | 8 | 54 | R | superior frontal |
| 24 | 3.53 | 3.20 | 45 | 23 | 11 | R | inferior frontal gyrus |
| 71 | 3.53 | 3.20 | -61 | -18 | 26 | L | postcentral |
| 13 | 3.44 | 3.13 | 46 | -62 | 18 | R | mid temporal |

**Table S8. Age-comparison of update bias (desirable update > undesirable update**). Uncorrected threshold p < 0.001, > 10 voxels.

| **No. voxels** | **T** | **Z** | **x** | **y** | **z** | **L/R** | **region** |
| --- | --- | --- | --- | --- | --- | --- | --- |
| **older > young** |  |  |  |  |  |  |  |
| 357 | 4.17 | 3.66 | 20 | -58 | 50 | R | superior parietal |
| 649 | 4.12 | 3.63 | 26 | -1 | -8 | R | putamen |
| 764 | 3.86 | 3.44 | -25 | -7 | -8 | L | putamen |
| 13 | 3.74 | 3.35 | 14 | -16 | 59 | R | supplementary motor area |
| 20 | 3.49 | 3.16 | 38 | -54 | 35 | R | angular gyrus |
|  |  |  |  |  |  |  |  |
| **young > older** |  |  |  |  |  |  |  |
| 1087 | 4.44 | 3.85 | 22 | -64 | -9 | R | lingual |
| 562 | 3.95 | 3.51 | -4 | -22 | 39 | L | mid cingulum |
| 217 | 3.86 | 3.44 | -54 | 15 | 26 | L | inferior frontal gyrus |
| 113 | 3.86 | 3.44 | -23 | -67 | -14 | L | fusiform |
| 205 | 3.83 | 3.42 | 9 | -98 | 21 | R | superior occipital |
| 102 | 3.72 | 3.33 | 4 | -80 | -26 |  | cerebellum |
| 53 | 3.69 | 3.31 | -8 | -99 | 21 | L | superior occipital |
| 28 | 3.63 | 3.27 | -27 | 25 | -34 | L | superior temporal pole |
| 62 | 3.60 | 3.24 | -2 | -68 | 20 | L | calcarine |
| 113 | 3.50 | 3.17 | 42 | -46 | -17 | R | fusiform |

**Table S9.** **Conjunction analysis of young > older and update undesirable young > update undesirable older.** Uncorrected threshold p < 0.001, > 10 voxels.

| **No. voxels** | **T** | **Z** | **x** | **y** | **z** | **L/R** | **region** |
| --- | --- | --- | --- | --- | --- | --- | --- |
| 514 | 4.21 | 3.70 | 62 | -38 | 22 | R | superior temporal |
| 356 | 3.96 | 3.52 | -7 | 38 | 45 | L | superior frontal |
| 76 | 3.64 | 3.29 | 54 | -59 | 21 | R | mid temporal |
| 16 | 3.63 | 3.28 | 19 | -43 | -21 | R | cerebellum |

**Table S10. Spearman’s correlations between anterior cingulate cortex (ACC) subregion grey matter volume and update bias.**

To account for the relatively small sample size, we also performed Spearman’s correlations for the correlation between dorsal & ventral subregions of anterior cingulate cortex (ACC) grey matter volume and update bias (desirable update minus undesirable update). The pattern of results remains the same as those using Pearson’s correlations as reported in Table 1.

rho = Spearman’s correlation coefficient (first value in cell) and partial Spearman’s correlation coefficient controlling for age, gender and total intracranial volume (second value in the same cell of the table). p = corresponding significance value.

|  | **Older** |  | **Young** |  |
| --- | --- | --- | --- | --- |
|  | **rho** | **p** | **rho** | **p** |
| **Dorsal ACC** | .833, .756 | .000, .001 | .086, .109 | .735, .699 |
| **Ventral ACC** | .643, .429 | .004, .110 | .084, .121 | .742, .667 |

**Table S10. Correlation coefficients (Pearson’s correlations) for the correlation between dorsal anterior cingulate cortex grey matter volume and desirable update and undesirable update.** r = Pearson’s correlation coefficient (first value in cell) and partial Pearson’s correlation coefficient controlling for age, gender and total intracranial volume (second value in the same cell of the table). p = corresponding significance value.

|  | **Young** |  | **Older** |  |
| --- | --- | --- | --- | --- |
|  | **r** | **p** | **r** | **p** |
| **Desirable** | .175, .229 | .488, .412 | .567, .415 | .014, .124 |
| **Undesirable** | .053, .258 | .836, .354 | -.134, -.178 | .596, .527 |

**Supplementary Material: List of stimuli presented to participants**

fraud when buying something on the internet

theft from vehicle

card fraud

victim of violence with need to go to A&E

sport related accident

household accident

mouse/rat in house

victim of violence by acquaintance

being cheated by husband/wife/partner

more than £30000 debts

miss a flight

witness a traumatising accident

domestic burglary

victim of violence by stranger

car/bicycle stolen

being convicted of crime

house vandalised

computer crash with loss of important data

skin burn

theft from person

victim of violence at home

having fleas/lice

severe injury due to accident (traffic or house)

victim of mugging

holiday cancelled due to natural disaster

public transport delay causing you to be late

identity fraud

insect infestation (e.g. ants) in your home

roof leak

spill difficult-to-remove substance (e.g. red wine) on carpet

short-changed in a shop

passenger in a car accident

lose your house keys

heating system in your house breaks down

stung by a bee

accidentally break something at a guests house

car vandalised

burn something you are cooking

serious disagreement with a good friend

receive unwanted call from telemarketer

shouted at by a stranger

more than 15 minutes late for an important meeting

bounce a cheque/payment

have a serious family argument

get a parking or speeding ticket

**Supplementary References**

1. Estévez-González A, Kulisevsky J, Boltes A, Otermín P, García-Sánchez C (2003): Rey verbal learning test is a useful tool for differential diagnosis in the preclinical phase of Alzheimer's disease: comparison with mild cognitive impairment and normal aging. *International Journal of Geriatric Psychiatry*. 18:1021-1028.

2. Beck AT, Ward CH, Mendelson M, Mock J, Erbaugh J (1961): An Inventory for Measuring Depression. *Arch Gen Psychiatry*. 4:561-571.

3. Strunk DR, Lopez H, DeRubeis RJ (2006): Depressive symptoms are associated with unrealistic negative predictions of future life events. *Behaviour Research and Therapy*. 44:861-882.

4. Weinstein ND, Klein WM (1995): Resistance of personal risk perceptions to debiasing interventions. *Health Psychology*. 14:132-140.

5. Helms G, Dathe H, Kallenberg K, Dechent P (2008): High-resolution maps of magnetization transfer with inherent correction for RF inhomogeneity and T1 relaxation obtained from 3D FLASH MRI. *Magnetic Resonance in Medicine*. 60:1396-1407.

6. Lutti A, Hutton C, Finsterbusch J, Helms G, Weiskopf N (2010): Optimization and validation of methods for mapping of the radiofrequency transmit field at 3T. *Magnetic Resonance in Medicine*. 64:229-238.

7. Alexander WH, Brown JW (2011): Medial prefrontal cortex as an action-outcome predictor. *Nat Neurosci*. 14:1338-1344.

8. Egner T (2011): Surprise! A unifying model of dorsal anterior cingulate function? *Nat Neurosci*. 14:1219-1220.
